# Influence of back squat eccentric tempo on countermovement jump performance and evaluation of PAPE detection thresholds

**DOI:** 10.1038/s41598-026-49865-6

**Published:** 2026-04-20

**Authors:** Miłosz Tchorowski, Artur Mohr, Beata Pożarowszczyk-Kuczko, Wiktoria Senator, Jarosław Domański, Dariusz Mroczek, Kamil Michalik

**Affiliations:** 1 Faculty of Physiotherapy, Wroclaw University of Health and Sport Sciences, Ignacego Jana Paderewskiego 35, Wroclaw, 51-612 Poland; 2 Praktyczna Strona Treningu, Katowice, Poland; 3 Faculty of Physical Education and Sports, Wroclaw University of Health and Sport Sciences, Wroclaw, Poland

**Keywords:** Resistance exercise, Jump performance, Muscle oxygenation, Performance enhancement, Conditioning activity, Health care, Medical research, Physiology

## Abstract

The aim of this study was to: (a) compare potentiation effect of three eccentric phase duration on countermovement jump (CMJ); (b) evaluate approaches for verification of post activation performance enhancement (PAPE); (c) verify whether muscle saturation and time of reoxygenation are relevant to explain PAPE. Eighteen men with experience in strength training completed four sessions. The first one included one repetition maximum testing (1RM) and familiarization. The remaining focused on potentiation protocol which was four repetitions with 80% 1RM at given tempo, 2/0/X/0 (FAST), 4/0/X/0 (MED), 6/0/X/0 (SLOW) respectively. Before and after conditioning activity (CA) CMJ was conducted. During CA barbell velocity and muscle saturation was monitored, There were no changes in CMJ height at 1, 4 and 8 min (F_(3153)_ = 0.65, *p* = 0.58) in each condition. However, for an approach with changes between pre-post analysis showed significant increases in the CMJ height for SLOW (3.5%, *p* < 0.05) and MED (4.2%, *p* < 0.01) The smallest worthwhile change analysis showed the highest proportion of participants exhibiting a PAPE effect. Muscle saturation did not differ tempo and time of reoxygenation has not any relationship on magnitude of PAPE. Methodological approach has a large influence on the examination PAPE. Muscle saturation does not provide explanation of PAPE.

## Introduction

In sport, especially in competitive setting, every opportunity is taken to gain an advantage over opponents. One effective method is application of a conditioning activity (CA) to induce post-activation performance enhancement (PAPE) during training or competition^1,2,8^. PAPE refers to a phenomenon in which a specific pre-activation exercise results in a rapid enhancement of neuromuscular performance^1,2^. The activating exercise typically involves high-intensity isotonic or isometric movements, leading to increased muscular output (i.e. strength and contraction speed). It is recommended that the CA movement pattern closely resembles the target task^3^. Strength exercises, such as performing high-load squats, are commonly used to elicit potentiation before explosive actions like countermovement jumps (CMJ)^4,5^.The proposed mechanisms underlying the PAPE effect include phosphorylation of myosin regulatory light chains, elevated muscle temperature, decreased muscle pH, increased blood flow, heightened muscle activity, motoneuron activation and discharge and enhances muscle and tendon stiffness^6^. Optimal management of fatigue i.e., rest time change may support the PAPE effect, but depending, among other factors on training level^7^– individualized approaches are necessary to optimize CA protocols.

Numerous previous studies have investigated the PAPE effect using different exercise modalities^8–11^, showing improvements in muscular performance ranging from 2,7% to 17%^9,12,13^, typically occurring between 4 and 12 min after the CA^12,14^. CAs have included various types of muscle contractions, including isometric^4,8,9,15^, concentric^4,12^ and eccentric movements^4,10,16^. Previous studies examined influence of different eccentric exercises on PAPE effect e.g. isoinertial^10^ or downward phase duration change^16^. Interestingly, movement tempo, a key variable in resistance training intensity^17^, has received relatively little attention. Based on study Wilk et al.^17^ movement tempo was classified as explosive (maximal speed), fast (1–2.9 s), medium (3–5.9 s), slow (6–9.9 s) and extremely slow (> 10 s). Bogdanis et al.^4^, for example, compared a maximal 3-second isometric contraction (ISO), a concentric contraction at 90% 1RM (CON) and an eccentric contraction at 70% 1RM (ECC) in half-squat exercise on CMJ performance. No PAPE effects was observed following the ECC protocol, although movement tempo was not manipulated. In contrast, Koźlenia and Domaradzki^15^ found that combining an isometric half-squat with maximal push intent and slow eccentric bodyweight squat resulted in CMJ improvement after 9 min. Similarly, Stastny et al.^16^ compared a fast eccentric phase in a front-loaded squat at 90% 1RM with a 2-second eccentric phase in a squat without the concentric component using 120% 1RM. Both protocols resulted in comparable PAPE effects, however prolonged eccentric duration (> 2 s) should be examine. Tsoukous et al.^18^ examined fast (2 s duration) and slow (6 s duration eccentric phase. He showed that tempo does not influence PAPE effect magnitude, even though in fast the neural drive was higher compared to slow. Mechanical response shown as mean velocity was also significantly higher in fast but as mentioned above PAPE did not differ. However, he used only bench press exercise, so the results cannot be generalized to every activity. Similarly Wilk et al.^19^ also examined bench press using fast (2 s duration) and slow (6 s duration) eccentric phase. He found that intentionally slowing down eccentric speed increase acute hormonal response. To our best knowledge no researcher examined the medium speed, which might be good compromise between increased fatigue (increased hormonal response) and decreased neural drive and mechanical response, which all are crucial in inducing PAPE effect. These justify further exploration of longer eccentric phases and their direct comparison in eliciting PAPE.

As previously mentioned, several variables influence PAPE magnitude by modulating fatigue levels. These include exercise volume, intensity, type of muscle action and recovery interval between CA and the subsequent explosive test^8^. Moreover, the assessment of PAPE varies: some studies consider absolute changes from baseline other use any positive percentage change, surpassing the smallest worthwhile change (SWC), standard error of measurement (SEM), coefficient of variation (CV) [1,7.18] or typical error^20^. It should be noted that not all individuals respond positively to CA, a distinction that continues to be investigated^7,20^. Monitoring exercise intensity and individual responses is essential for tailoring CA protocols to athletes specific needs. Thus, an interesting approach seems to be an exploratory examination of participants whose observed changes exceeded measurement error thresholds.

Various approaches have been used to assess CA intensity in strength training research. These include relative load (%1RM)^21^, number repetitions and sets^22^, velocity loss in velocity-based training (VBT)^23^ and rest duration between CA and testing^14^. Some authors also advocate using the “reps in reserve” (RIR) method^21,24^. A modern technique for monitoring physiological responses is near-infrared spectroscopy (NIRS), non-invasive method for measuring local blood oxygen saturation using non-infrared light^25^. This allows for real-time tracking of physiological responses^26^. NIRS has already been used in sports to monitor changes in muscle oxygen saturation (SmO_2_) during endurance and resistance training^27^, as blood volume and intramuscular oxygenation may vary between muscles^28^. Moreover, different types of muscle contractions elicit distinct oxygenation patterns^29^. This real-time feedback allows researchers to understand how training variables such intensity, volume, rest intervals and tempo influence on local muscle saturation and acute responses during CA^6,26^. Additionally, NIRS enables the assessment of mitochondrial oxidative capacity which is critical during high-intensity efforts^30^, due to the link between reoxygenation time and phosphocreatine (PCr) resynthesis kinetics^31^. A shorter hemoglobin reoxygenation time may positively contribute to PAPE effect. Thus, monitoring both immediate and prolonged post-CA responses may help optimize and individualize PAPE protocols.

Based on the above, the first aim of this study was to compare effects of different eccentric phase durations (2,4 and 6 s)^17^ during barbell squats on countermovement jump (CMJ) performance. The second aim was to evaluate various approaches for verifying the occurrence the highest proportion of participants exhibiting a PAPE effect (exploratory examination). The third aim was to determine whether monitoring muscle oxygen saturation may be useful for assessing the intensity of the conditioning activity (CA) and whether the rate of reoxygenation is associated with achieving the PAPE effect. We hypothesize that all protocols will result in improved CMJ performance although longer eccentric phases are likely to induce superior results.

## Methods

### Participants

The study involved 18 healthy, physically active young men. All participants were volunteers who reported a minimum of two years of resistance training experience and no musculoskeletal injuries in the six months prior the study. None of the participants were engaged in professional-level sports. All recruited participants were informed about the study procedures, provided written informed consent and completed the study protocol. The study was approved by the local Research Ethics Committee (approval number 29/2024) and conducted with the Declaration of Helsinki. Detailed characteristics of the participants are presented in Table 1.

The G*Power 3.1 software (v.3.1.9.2, Heinrich-Heine-Universität Düsseldorf, Dusseldorf, Germany) was used to determine the sample size a priori^32^. The expected effect size (partial eta-square - η^2^) based on previous study^16^, was set at 0.14, the α level was set at 0.05, and the power (1-β) was set at 0.90^33^. Eighteen participants in the group were necessarily recruited.

**Table 1:** Participants’ characteristics.

Variable	x̄±SD	95% CI
Age (years) Height (cm) Body mass (kg) BMI (kg·m^− 2^) Resistance training experience (years) PA (hours per week) 1RM (kg) 1RM/ body mass	22.2 ± 2.1 180 ± 7 82.8 ± 9.7 25.3 ± 2.6 6.2 ± 3.0 7.3 ± 2.4 135.4 ± 22.6 1.7 ± 0.2	21.2–23.3 176–183 77.0-86.6 24.0-26.6 4.7–7.7 6.1–8.5 124.2-146.6 1.5–1.8

BMI – body mass index, PA – self reported physical activity, 1RM – one repetition maximum in back squat.

### Experimental protocol

**Fig. 1:**
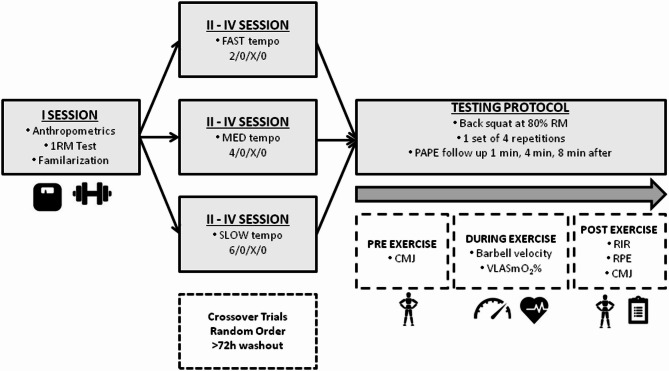
Experimental protocol.

The participants attended the gym on four separate occasions with 72-hour intervals between sessions each conducted at the same time of day (Fig. 1). They were instructed to refrain from intense physical activity for 24 h prior to each session. The first visit included measurements of height (cm) and body mass (kg) using medical scale (WPT 200, Radwag, Radom, Poland) and familiarization session was conducted to acquaint participants with the study procedures. During this visit participants performed a one-repetition maximum (1RM) back squat test and were introduced to correct technique of the CMJ protocol and squats with varying eccentric tempos (one set of four back squat repetitions performed at 80% 1RM for each tempo with 5 min of passive recovery between sets). During sessions 2 to 4 participants underwent eccentric conditioning activities for CMJ potentiation. Eash session involved one set of four back squat repetitions at 80% 1RM as CA using one of the following eccentric tempos: 2/0/X/0 (FAST), 4/0/X/0 (MED) or 6/0/X/0/ (SLOW)^17^ with X meaning maximal concentric velocity. Two CMJs were performed after general warm-up and the best scores from three sessions were used to assess individual jump height variability. After completing a standardized warm-up with a barbell, participants performed a CMJ again which served as the baseline measurement (CMJ_PRE_). During the CA the average barbell velocity in the concentric phase was monitored using a linear position transducer and muscle oxygen saturation of the vastus lateralis (VLA) was measured. Following the CA participants reported their perceived exertion using RPE and RIR scales and after seated 1 min break performed CMJ tests at 1 (CMJ_1_), 4 (CMJ_4_) and 8 min (CMJ_8_) post-CA to assess the presence of the PAPE effect. During the intervals between jumps participants rested passively.

### One-repetition maximum test (1RM test)

Prior to testing each participant completed a warm-up consisting of 5 min of unloaded cycling on cycle ergometer. The 1RM determination protocol included five preparatory sets with approximate loads of 20% 1RM (3 repetitions), 40% 1RM (3 repetitions), 60% 1RM (3 repetitions), 80% 1RM (1 repetition) and 90% 1RM (1 repetition) followed by the first 1RM attempt. The back squat was performed to IPF depth standards, assessed by the investigator, with foot placement corresponding to the participants shoulder-width stance, and with accordance to others IPF standards^34^. Participants were allowed up to five 1RM attempts while maintaining proper technique. A 3-minute passive rest interval was provided between attempts. The use of supportive equipment such as belts, knee wraps or weightlifting shoes was not permitted.

### CMJ height assessment

Countermovement jump (CMJ) height was measured using the OptoGait system (MicroGate, Bolzano, Italy). Each CMJ was initiated from a standing position between two optical bars with the knees fully extended and hand places in the hips to eliminate the influence of arm swing. Participants were instructed to lower themselves to a self-selected depth and then jump as high and as quickly as possible. Previous studies reported excellent reliability with ICCs > 0.80 and the CV < 10% for CMJ height for photocells system^35^.

### PAPE stimulation

Sessions 2 to 4 began with standardized 10-minute warm-up (5 min on cycle ergometer followed by 5 min of mobility exercises) This was followed by back squats with loads of 20% 1RM (3 repetitions), 40% 1RM (3 repetitions) and 60% (3 repetitions) performed at the designated movement tempo with a 3 min rest between sets. The conditioning activity consisted of a single set of four back squat repetitions 80% 1RM using predetermined eccentric tempo (FAST, MED, SLOW as described above). The order of tempo execution in each session was randomized using an online generator (www.randomizer.org). The tempo was controlled and guided by the researcher using a metronome. Squats were performed in same form as described above.

### Barbell velocity measurement

During each repetition of the squat the mean concentric velocity of the barbell was monitored using a linear position transducer ICC = 0,98, CV = 8,5%^36^(Tendo Sport Machines, Trencin, Slovakia). The device was placed on the floor centrally aligned with the starting position of the barbell and connected via a tether. The setup was positioned perpendicular to the ground to minimize the influence of horizontal movement on the measurements. Mean velocity (VEL_mean_) and velocity loss (VL) during the set were calculated.

### Muscle saturation (SmO_2_)

During the test, a near-infrared spectroscopy (NIRS) device (Moxy Monitor; Fortiori Design LLS, Hutchinson, MN, USA) ICC = 81, CV = 6%^37,38^, was placed on VLA. The sensor was positioned 15 cm above the superior border of the patella approximately on middle part of VLA muscle belly and secured with black adhesive tape. The following parameters were included in the analysis: resting saturation one minute before the conditioning activity (SmO_2_ rest), the minimum saturation value during the exercise (SmO_2_ min), the difference between SmO_2_ rest and SmO_2_ min (SmO_2_ Δdeoxy) and the time required to reach 50% reoxygenation following the last repetition (tSmO_2_ 50%reoxy).

### Perceptual responses (RPE, RIR)

After completing the test exercise participants reported their perceived exertion using the RPE scale and indicated the number of repetitions they believed they could still perform (RIR). A modified Borg scale (0–10)^39^ was used to express subjective effort, while the RIR scale reflected the number of additional repetitions the participant estimated they could complete if the set were not terminated.

### Statistical analysis

For statistical analysis we used the IBM SPSS Statistics version 26 software package (IBM, Inc., Chicago, IL, USA). The results are presented as mean ± standard deviation (x̄±SD) and 95% confidence intervals (95% CI). The normality of the distribution was evaluated by the Shapiro–Wilk test and homogeneity of variance was assessed using Levene’s test. One-way analysis of variance (ANOVA) was used to compare differences in acute responses between movement tempo (VL, RPE, RIR, SmO_2_ rest, SmO_2_ min, SmO_2_ Δdeoxy) and absolute difference in CMJ height (DIFF). Analysis of variance with repeated measures (RM-ANOVA) *tempo* x *time* was performed for examination of barbell velocity during consecutive repetitions and for CMJ after CA with different tempo. The Bonferroni post-hoc test was performed when a significant F ratio was obtained. Sphericity was examined using Mauchly’s test, and Greenhouse–Geisser corrections were applied where appropriate. The non-parametric Kruskal-Wallis test was used for further analysis of mean velocity and time for tSmO_2_ 50%reoxy. The effect size was calculated as partial eta-square (η^2^) (small ≥ 0.01 to ≤ 0.06, moderate ≥ 0.07 to ≤ 0.13, and large ≥ 0.14). The Spearman’s rank correlation coefficient was calculated between tSmO_2_ 50%reoxy and CMJ height and DIFF in the first minute after CA. The intra-rater reliability analysis involved CV, SEM, SWC and minimal detectable change (MDC) was performed to assess the consistency of CMJ height measurements across different days during the entire study period. To determine the practical and statistical significance of the PAPE effect for the absolute difference in CMJ height the SWC and MDC threshold were examined. CV was calculated from the following equation: SD/mean·100. SEM was calculated as follows: SEM = SD/√3. SWC was calculated by multiplication SD by 0.2. MDC was derived from equation: MDC = SEM·1.96·√2. An individual change was classified as exceeding measurement error when the difference across variables was greater than the appropriate indicator (i.e. daily variation). The *p* ≤ 0.05 level was considered as statistically significant.

## Results

**Table 2:** Barbell velocity during four back squat repetitions (x̄±SD and 95% CI).

Tempo	Rep 1 (m/s)	Rep 2 (m/s)	Rep 3 (m/s)	Rep 4 (m/s)
FAST	0.62 ± 0.08 (0.58–0.66)	0.59 ± 0.08* (0.54–0.63)	0.56 ± 0.09*** (0.52–0.61)	0.54 ± 0.09*** (0.50–0.59)
MED	0.60 ± 0.09 (0.55–0.64)	0.57 ± 0.09* (0.52–0.61)	0.54 ± 0.11*** (0.48–0.60)	0.52 ± 0.13*** (0.45–0.58)
SLOW	0.59 ± 0.08 (0.55–0.63)	0.55 ± 0.09 (0.51–0.59)	0.54 ± 0.10*** (0.49–0.58)	0.51 ± 0.10*** (0.46–0.55)

Significant difference between first repetition: * - *p* < 0.05, ** - *p* < 0.01, *** - *p* < 0.001.

The RM-ANOVA revealed significant the main effect for *time* (F_(3,153)_ = 82.9, *p* < 0.001, η^2^ = 0.62), but not for *tempo* (F_(2,51)_ = 0.60, *p* = 0.55, η^2^ = 0.02) and interaction *tempo* x *time* (F_(6,153)_ = 0.34, *p* = 0.91, η^2^ = 0.01). The Bonferroni post hoc test showed decreasing barbell velocity for FAST, MED and SLOW in consecutive repetitions (Table 2).

**Table 3:** Mechanical, perceptual and physiological responses to different tempo during back squat exercise (x̄±SD and 95% CI).

Variable	FAST	MED	SLOW	Analysis of variance		
				F	*p*	η^2^
VEL mean (m/s)	0.58 ± 0.08 (0.54–0.62)	0.55 ± 0.10 (0.50–0.61)	0.54 ± 0.09 (0.50–0.59)	0.60	0.55	0.02
RPE (AU)	5.4 ± 2.1 (4.3–6.4)	6.4 ± 1.8 (5.5–7.3)	6.7 ± 1.9 (5.7–7.6)	2.18	0.12	0.08
RIR (AU)	4.0 ± 1.6 (3.2–4.8)	2.8 ± 1.4^*^ (2.1–3.5)	2.8 ± 1.1^*^ (2.2–3.3)	4.85	0.01	0.16
SmO_2_ rest (%)	61.0 ± 6.6 (57.7–64.3)	63.4 ± 8.2 (59.4–67.5)	65.4 ± 8.8 61.0-69.7)	1.38	0.26	0.05
SmO_2_ min (%)	22.7 ± 12.6 (16.5–29.0)	20.8 ± 11.2 (15.2–26.4)	20.6 ± 13.6 (13.8–27.4)	0.16	0.85	0.01
SmO_2_ Δdeoxy (%)	38.2 ± 8.7 (33.9–42.6)	42.6 ± 11.2 (37.1–48.2)	44.8 ± 13.6 (38.4–51.1)	1.63	0.20	0.06

VEL mean – mean barbell velocity during back squat, RPE – rate of perceived exertion, RIR – reps in reserve, SmO_2_ rest – baseline muscle saturation, SmO_2_ min – the lowest muscle saturation, SmO_2_ Δdeoxy - percent change of muscle saturation from baseline * – significant difference between FAST.

Table 3 displays mechanical, perceptual and physiological responses to different CA. One-way ANOVA with Bonferroni post hoc test showed that only in RIR was significant difference and greater value was observed in FAST compared to MED (*p* < 0.05) and SLOW (*p* < 0.05). The Kruskal-Wallis test did not reveal any significant differences (H = 0.69, df = 2, *p* = 0.71) between FAST (12.2 ± 5.1, 95% CI 9.7–14.7%), MED (14.4 ± 11.1, 95% CI 8.9–20.0%) and SLOW (14.2 ± 6.9, 95% CI 10.7–17.6%) for velocity loss (VL) during performing back squat. Time to recovery 50% of baseline value (tSmO_2_ 50%reoxy) did not differ between tested conditions (H = 3.68, df = 2, *p* = 0.16) and equaled 21.7 ± 6.9 (95% CI 18.2–25.1) (s), 25.5 ± 9.4 (95% CI 20.8–30.2) (s), 27.1 ± 9.7 (95% CI 22.3–31.9) (s) for FAST, MED, SLOW, respectively.

There were not found any main effects in CMJ height for *time* (F_(3,153)_ = 0.65, *p* = 0.58, η^2^ = 0.01), *tempo* (F_(2,51)_ = 0.01, *p* = 0.99, η^2^ = 0.0004) and interaction *tempo* x *time* (F_(6,153)_ = 1.39, *p* = 0.23, η^2^ = 0.05) (Table 4).

**Table 4:** Countemovement jump before and after PAPE stimulation (x̄±SD and 95% CI).

Tempo	CMJ_PRE_ (cm)	CMJ_1_ (cm)	CMJ_4_ (cm)	CMJ_8_ (cm)
FAST	41.4 ± 5.9 (38.5–44.3)	41.6 ± 6.4 (38.4–44.8)	41.5 ± 5.3 (38.8–44.1)	40.6 ± 6.2 (37.6–43.7)
MED	41.2 ± 5.9 (38.3–44.1)	42.1 ± 5.6 (39.3–44.9)	41.3 ± 5.9 (38.4–44.3)	41.6 ± 6.2 38.5–44.7)
SLOW	41.1 ± 5.8 (38.2–44.0)	41.1 ± 6.0 (38.1–44.1)	41.6 ± 5.7 (38.7–44.4)	41.6 ± 5.7 (38.8–44.5)

**Fig. 2:**
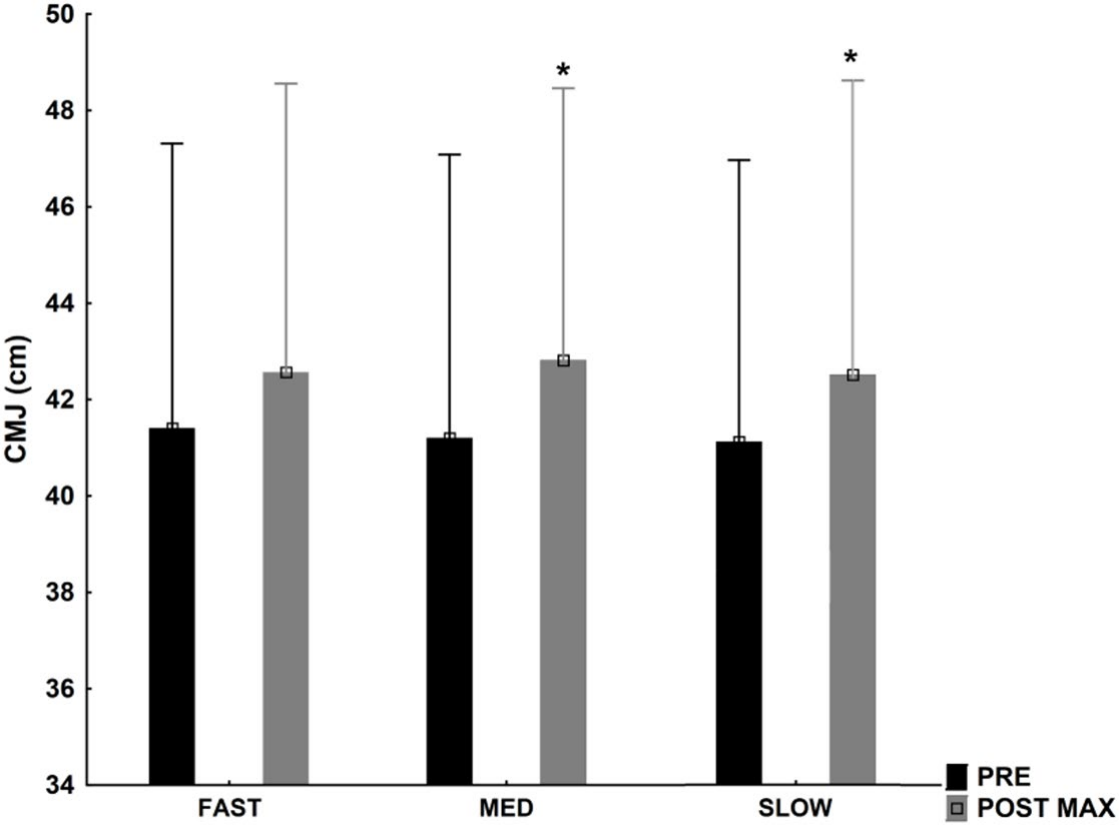
Comparison of CMJ height (x̄ ± SD) between pre and post (the highest value) for FAST, MED and SLOW tempo. * - significant difference between pre and post.

Taking into account approach with changes between pre and post (the highest CMJ value) the RM-ANOVA revealed the main effect for *time* (F_(1,51)_ = 30.09, *p* < 0.001, η^2^ = 0.37), but not for *tempo* (F_(2,51)_ = 0.01, *p* = 0.99, η^2^ = 0.0002) and interaction *tempo* x *time* (F_(2,51)_ = 0.26, *p* = 0.77, η^2^ = 0.01). Bonferroni post hoc analysis showed significant increases in the CMJ height between pre and after conditioning activity for SLOW (3.5 ± 5.7%; 95% CI 0.7–6.3; *p* < 0.05) and MED (4.2 ± 4.9%; 95% CI 1.7–6.6; *p* < 0.01), but not for FAST (2.9 ± 3.2%; 95% CI 1.3–4.5; *p* = 0.16) (Fig. 2). The one-way ANOVA revealed that absolute differences between three conditions were not significant (F_(2,51)_ = 0.26, *p* = 0.77, η^2^ = 0.01) and equaled 1.2 ± 1.3 (95% CI 0.5–1.8) (cm) (DIFF_FAST_), 1.6 ± 1.9 (95% CI 0.7–2.5) (cm) (DIFF_MED_) and 1.4 ± 2.3 (95% CI 0.3–2.5 (cm) (DIFF_SLOW_). The highest individual percentage improvement for FAST, MED and SLOW were 8,4%, 14,4%, 11,5% respectively.

From a statistical and practical point of view, there were analyzed absolute differences in CMJ height between pre and post for three CA in regards to daily variability. The magnitude of DIFF_MED_ and DIFF_SLO_ were greater compared to SEM (0.9 ± 0.6 cm; 95% CI 0.6–1.2) and SWC (0.3 ± 0.2 cm; 95% CI 0.2–0.4), but lower than MDC (2.6 ± 1.6 cm; 95% CI 1.7–3.4). Further analysis showed that percentage improvement of CMJ height was higher than CV (3.9 ± 2.4%; 95% CI 2.7–5.1) only for MED tempo. The number of individual changes was determined by identifying absolute or percentage differences that exceeded the measurement error and was shown in Table 5.

**Table 5:** Number of individual changes for reliability indicators: coefficient of variation (CV), standard error of measurement (SEM), the smallest worthwhile change (SWC) and minimal detectable change (MDC).

Tempo	CV (*n*)	SEM (*n*)	SWC (*n*)	MDC (*n*)
FAST	7	12	15	3
MED	8	12	15	4
SLOW	9	13	14	5

There were no significant rank Spearman’s correlations between tSmO_2_ 50%reoxy and CMJ_1_ after CA in FAST (rho = -0.11), MED (rho = 0.17), SLOW (rho = 0.06). Also, lack of significant correlations occurred between tSmO_2_ 50%reoxy and DIFF_FAST_ (rho = -0.25), DIFF_MED_ (rho = -0.04) and DIFF_SLOW_ (rho = 0.01).

## Discussion

This study investigated: (i) the effect of different eccentric phase durations during the squat on CMJ performance; (ii) exploratory responders analysis of selected approaches for verifying the PAPE effect and identifying individual changes and (iii) the usefulness of muscle oxygen saturation measurements for assessing the intensity of CA and the impact of reoxygenation rare on the magnitude of the PAPE effect. Our primary analysis did not confirm our hypothesis, as no significant group-level PAPE effects were observed at 1, 4–8 min post-CA. When an exploratory, post-hoc analysis based on the highest CMJ value achieved regardless of time point was applied, a significant main effect of time was observed; however, no main effect of tempo or tempo × time interaction was detected. The exploratory analysis further revealed that the number of individual changes classified as exceeding reliability thresholds varied depending on the selected indicator. Simultaneously muscle oxygen saturation showed a non-significant trend toward greater deoxygenation with longer eccentric phases and the tSmO_2_ 50% reoxygenation time did not influence the magnitude of the PAPE response.

To our knowledge this is one of the first studies comparing the effects of different eccentric phase durations (with 80% 1RM load) during CA on CMJ performance. Previous research has examined movement tempos but not in direct comparison with one another – instead comparing tempo-based interventions to control conditions^15,16^. Only Tsoukos et al.^18^ compared the effects of fast and slow tempos (both eccentric and concentric) during the bench press on PAPE in the bench press throw. They demonstrated the effectiveness of both approaches assuming equal time under tension^18^. We observed no significant effects of tempo, time, or their interaction in our primary analysis. An exploratory analysis examining the highest post-CA jump (regardless of timing) suggested small increases in CMJ height following MED (4.2%) and SLOW (3.5%) protocols; however, these did not differ significantly from FAST (2.9%), and the analysis was not pre-planned. These findings suggest that all three tempo conditions may produce modest, similar potentiation effects rather than meaningful tempo-dependent differences. Contrary to our findings, Statsny et al.^16^ reported a significant PAPE effect following fast (2 s) eccentric phase; however, they used CA at 120% 1RM (eccentric-only), whereas we used 80% 1RM with a concentric phase. The absence of clear group-level PAPE effects in our primary analysis may be partly explained by insufficient stimulus intensity in the applied conditioning activity. In the present study, a load of 80% 1RM was selected to allow strict control of eccentric tempo across conditions while limiting excessive fatigue and ensuring technical consistency. However, this choice likely resulted in a stimulus that was lower than that typically used in PAPE research, where loads of ≥ 85% 1RM are most commonly reported to induce robust potentiation responses. This methodological decision therefore represents an important limitation and may partly account for the null findings observed at the group level.

Previous studies have demonstrated that faster tempos are associated with greater sEMG activity and higher neural drive^18^, whereas a prolonged eccentric phase may increase local muscle temperature and muscle–tendon stiffness, both of which have been proposed as contributors to PAPE mechanisms^6^. Whereas faster eccentric actions are typically associated with greater neural drive, slow eccentric phases performed at submaximal loads may allow force production with lower motor unit activation, thereby reducing the recruitment of high-threshold motor units, particularly type II fibres, which are central to potentiation responses^40,41^. In our protocol, the concentric phase was performed with maximal intended velocity, which may have partially compensated for the lower external load by increasing neural drive. This combination of prolonged eccentric duration with maximal concentric intent may help explain the small pre–post improvements observed in the exploratory analysis despite the relatively moderate load. Nevertheless, higher external loads (≥ 85% 1RM), greater conditioning volumes, or multi-set protocols may be necessary to elicit more robust and time-dependent PAPE effects.

As noted earlier, our individual analysis indicated that the PAPE effect occurred at different time points across participants, highlighting the individualized nature of PAPE responses, which may be influenced by training experience – consistent with previous reports^5,7,20^. Trained individuals often require shorter recovery periods post-CA and may exhibit stronger PAPE effects than less experienced individuals^12^. Therefore, the exploratory examination of individual changes is of particular interest. We used several commonly applied indicators^1,2,7,20^. Absolute changes in jump height were compared against SWC, SEM and MDC thresholds while percentage changes were related to CV. Our exploratory responder analysis demonstrated that threshold choice substantially affects interpretation, with 67–72% exceeding SWC versus 17–28% exceeding MDC. However, our design cannot determine whether these represent stable individual characteristics. This is not surprising as SWC, a practically useful threshold, can detect subtle improvements but carries a higher risk of false positives. In contrast, MDC is a conservative threshold; surpassing it provides strong evidence for a genuine PAPE effect beyond random variation or measurement error^42,43^. Ultimately the methodological debate about the existence of the PAPE effect remains valid, particularly given that even minimal performance gains can be critical in elite sport. Sport scientists should consider to replicate crossover designs, in which participants complete multiple cycles of each condition, would be required to quantify true person by treatment variance and to identify genuine differential responders.

To better contextualise the conditioning activity, mechanical, physiological, and perceptual responses were monitored. Only RIR differed significantly between tempos; however, RPE and mean velocity showed similar directional patterns, with slower eccentric conditions tending toward higher perceived effort and lower barbell velocity. Given that our a priori power analysis was based on a large assumed effect size, the study may have been underpowered to detect smaller tempo-related differences in these variables. The converging trends across RIR, RPE, and velocity suggest that slower eccentric tempos likely increased overall effort, which should be acknowledged as a potential confounding factor. Accordingly, any exploratory performance changes cannot be attributed to eccentric duration per se, but may instead reflect greater concentric demands imposed by the slower conditions. Velocity loss values were similar across conditions (~ 10–15%), aligning with previous work suggesting VL thresholds of ~ 10%^44^ or ~ 20%^45^ to support potentiation-oriented protocols, whereas Yuan et al.^46^ observed greater CMJ potentiation with ~ 5% VL when using higher loads (85% 1RM). Together, these findings suggest that regulation of effort and volume (e.g., via RIR and VL), rather than eccentric tempo alone, may be more relevant to PAPE expression and warrants further investigation.

A novel feature of our study was the assessment of muscle oxygenation. Interestingly, local SmO_2_ in the vastus lateralis did not differentiate between movement tempos, although a trend toward increased SmO_2_ Δdeoxy was observed with longer eccentric durations. Additionally, our findings do not support the idea that faster hemoglobin reoxygenation contributes to a greater PAPE response. The previously proposed mechanism linking reoxygenation rate to PCr resynthesis kinetics^31^ appears to have limited practical relevance here with neuromuscular factors likely playing a more significant role^6^. Since both reoxygenation time and RIR failed to differentiate between the MED and SLOW conditions. The MED protocol may be favored due to its greater amplitude of CMJ improvement and shorter execution time.

Given the absence of tempo-dependent differences in our primary analysis, we cannot confidently recommend one eccentric tempo over another for PAPE induction. If practitioners wish to explore eccentric-based CA protocols, our exploratory findings suggest that 4–6 s eccentric phases combined with maximal concentric intent at 80% 1RM may be worth investigating, targeting RIR ~ 3 and velocity loss < 15%. However, several important caveats apply: Our primary analysis showed no significant PAPE effects at any timepoint. Only 17–28% of participants exceeded MDC thresholds (our most conservative criterion). The choice of 80% 1RM may have been insufficient; loads ≥ 85% 1RM are more commonly used Individual responses varied considerably and may not be repeatable Future research should examine higher loads and greater CA volumes before definitive recommendations can be made.

This study has several limitations. First, the use of 80% 1RM may have been insufficient to elicit robust PAPE effects, as most previous studies have applied loads ≥ 85% 1RM, which may partly explain the lack of clear group-level effects. Second, our a priori power analysis assumed a large effect size (η²=0.14); therefore, the study was likely underpowered to detect small-to-moderate differences between similar tempo conditions, as reflected by the non-significant but consistent trends observed for RPE and mean velocity. Third, although the crossover design was appropriate for analysing mean differences, the single-cycle structure does not allow true response heterogeneity to be assessed; thus, the individual-level findings should be considered exploratory and require confirmation using replicate crossover designs^47^.In addition, slower eccentric tempos appeared to necessitate greater concentric effort, as indicated by RIR, RPE trends, and velocity patterns, which represents a potential confounding factor when interpreting eccentric-specific effects. The exploratory “best post-conditioning jump” analysis was not pre-planned and involved multiple testing without formal alpha adjustment, and should therefore be interpreted cautiously. Further limitations include the relatively small sample size, the absence of a self-selected eccentric tempo condition, and the use of only a single conditioning set. Future studies should investigate higher loads and greater conditioning volumes to better characterise both group-level and individual PAPE responses.

## Conclusions

Our primary analysis revealed no significant effects of eccentric tempo duration (2, 4, or 6 s) on CMJ performance at 1, 4, or 8 min following low-volume (4 reps, 80% 1RM) back squat conditioning activity. Exploratory post-hoc analysis suggested small improvements (2.9–4.2%) when examining the highest post-CA jump regardless of timing, but these did not differ significantly between tempo conditions. The proportion of participants exceeding reliability thresholds varied substantially based on criterion choice (SWC: 67–72%; MDC: 17–28%), highlighting the importance of threshold selection in PAPE research. However, our single-cycle design cannot determine whether these represent stable individual response patterns or random variation. We do not recommend muscle oxygen saturation monitoring as a method for predicting PAPE magnitude, as reoxygenation time showed no relationship with performance changes. Future research should examine higher CA loads (≥ 85% 1RM), greater training volumes, and employ replicate crossover designs to properly assess individual response heterogeneity to eccentric tempo variations.

## Data Availability

The datasets generated during the current study are not publicly available due to restrictions imposed by the local ethics committee and the terms of participant informed consent, which did not include public deposition of data in open repositories. The data contain detailed physiological and performance profiles which, in combination, may permit indirect identification of participants within a relatively small population. Fully de-identified datasets are available from the corresponding author (contact: milosztchorowski@gmail.com)upon reasonable request, subject to approval by the institutional ethics committee and the establishment of an appropriate data-sharing agreement.
